# Molecular mechanisms of mitochondria-mediated ferroptosis: a potential target for antimalarial interventions

**DOI:** 10.3389/fcell.2024.1374735

**Published:** 2024-04-10

**Authors:** Adegbolagun Grace Adegboro, Israel Sunmola Afolabi

**Affiliations:** ^1^ Department of Biochemistry, College of Science and Technology, Covenant University, Ota, Nigeria; ^2^ Covenant Applied Informatics and Communication Africa Centre of Excellence (CApIC-ACE), Covenant University, Ota, Nigeria

**Keywords:** ferroptosis, mitochondria, iron, antimalarial drug resistance, reactive species, antiparasitic

## Abstract

Ferroptosis is an iron-dependent form of regulated cell death characterized by glutathione (GSH) depletion, glutathione peroxidase 4 (GPX4) inactivation, and the build-up of lipotoxic reactive species. Ferroptosis-targeted induction is a promising therapeutic approach for addressing antimalarial drug resistance. In addition to being the primary source of intracellular energy supply and reactive oxygen species (ROS) generation, mitochondria actively participate in diverse forms of regulated cell death, including ferroptosis. Altered mitochondrial morphology and functionality are attributed to ferroptosis. Diverse mitochondria-related proteins and metabolic activities have been implicated in fine-tuning the action of ferroptosis inducers. Herein, we review recent progress in this evolving field, elucidating the numerous mechanisms by which mitochondria regulate ferroptosis and giving an insight into the role of the organelle in ferroptosis. Additionally, we present an overview of how mitochondria contribute to ferroptosis in malaria. Furthermore, we attempt to shed light on an inclusive perspective on how targeting malaria parasites’ mitochondrion and attacking redox homeostasis is anticipated to induce ferroptosis-mediated antiparasitic effects.

## 1 Introduction

Malaria persists as a menace to humanity, affecting several hundred million people and causing roughly 608,000 deaths in 2022 (WHO, 2023). The burden of malaria in the world is still excessively heavy in the WHO African Region. About 94% of malaria cases and 95% of mortality from malaria occurred in the region in 2021, with 78% emanating from children under the age of five (WHO, 2023). The World Health Organization’s (WHO) Global Malaria Program’s (GMP) main objectives are to control and eradicate malaria. To accomplish this, the WHO suggests the use of antimalarial medications; however, current reports of resistance to frontline antimalarial therapies such as artemisinin-based combination therapies (ACTs), as well as the lack of a 100% efficacious vaccine, have limited the eradication of malaria in high-burden countries (Phillips et al., 2017; Sena-dos-Santos et al., 2021). Various research has delved into the innate constituents of the host, such as genome assessment and understanding the mode of action of the body’s immune system, to find novel ways to combat malaria. However, various stages are involved in *Plasmodium* infection; hence, non-susceptibility to malaria must be encompassing with distinctiveness in the phases. Cell death is one of the immune system’s defenses against *Plasmodium* (Sena-dos-Santos et al., 2021).

Ferroptosis is typically a monitored cell death other than apoptosis that is initiated by iron-dependent oxidative stress and the oxidative degradation of lipids (Zhang et al., 2022). In contrast to apoptosis, which the usual cellular metabolism can initiate, ferroptosis is more related to internal stressors like oxidative stress and other metabolic dysregulations without the involvement of typical pro-death proteins associated with apoptosis (Dixon et al., 2012). Additionally, it differs from other regulated cell deaths in terms of its morphology and underlying mechanisms. Diverse processes, including iron, energy, lipid metabolisms, and specific small molecules, influence the course of ferroptosis and the susceptibility of cells to the process (Feng et al., 2023). Given the emerging understanding of ferroptosis, it has been implicated in the pathogenesis of malaria. The proof entails plasmodium-induced biochemical alterations that can influence the parasites and host red blood cells’ susceptibility to ferroptosis as well as the involvement of ferroptosis in decreasing parasite viability at the liver stage (Gowda and Wu, 2018; Kain et al., 2020; Sena-dos-Santos et al., 2021). Additionally, the potency of the frontline antimalarial compound dihydroartemisinin against both cancer and *Plasmodium* is attributed to its involvement in ferroptosis-mediated processes (Du et al., 2019; Li et al., 2022; Dokunmu et al., 2023). According to Huang et al. (2022), ferroptosis was implicated in the significant growth inhibition of *Toxoplasma gondii*, an apicomplexan parasite.

In addition to the mitochondria being the major source of intracellular energy, they also play a role in numerous physiological and pathological activities (Huang et al., 2020; Liu et al., 2023). Basically, mitochondria mediate the various forms of regulated cell death, including apoptosis, pyroptosis, necroptosis, and ferroptosis (Gao et al., 2019; Bock and Tait, 2020; Li et al., 2021a; Weindel et al., 2022). In ferroptosis, mitochondria undergo a morphological alteration, such as high membrane density and diminished mitochondrial cristae (Gao et al., 2019). Furthermore, mitochondrial energy metabolism is altered in ferroptosis; the generation of oxidative phosphorylation and energy (ATP) are typically elevated, while glycolysis is decreased (Wang et al., 2020a; Liu et al., 2023). Additionally, the oxidative stress level is elevated, and limitless oxidative stress results in unalterable damage to mitochondria, reducing their integrity and eventually resulting in energy diminution and cell death.

The induction of ferroptosis in malaria parasites depicts hugely promising potential for combating drug-resistant malaria parasites. Diverse ferroptosis inducers, such as erastin, were identified and recently acknowledged as a novel malaria-eradicating, potent target. The robust understanding of ferroptosis and its intracellular implications may bring about the identification of new and more effective therapeutic targets for drug-resistant malaria parasites. The numerous functions of mitochondria will be explained to showcase the association between mitochondria and ferroptosis, which aids in gaining deeper insights into the significance of ferroptosis to mitochondria in malaria parasites.

## 2 Mechanisms of ferroptosis

Ferroptosis is set off when there is a dysregulation in intracellular iron, leading to an excessive build-up of lipotoxic reactive oxygen species that overwhelm the free radical scavenging ability of the cell, hence disrupting the membrane structure and leading to cell death (Tao et al., 2020). The Nomenclature Committee on Cell Death described ferroptosis as an oxidative reaction of the intracellular milieu controlled by the enzyme glutathione peroxidase 4 (GPX4) as well as a type of controlled cell death that can be restrained by iron chelating agents and lipid-dissolving antioxidants (Galluzzi et al., 2018).

### 2.1 Hallmarks of ferroptosis

The earliest recognition of certain small molecules that brought about cell death other than apoptosis led to the emanation of ferroptosis, which was discovered to be controlled by iron (Fe^2+^) and mediated by disruption in normal molecular processes (Stockwell, 2022). Additionally, ferroptosis brings about a different mitochondrial membrane morphology from other classes of cell death (Dixon et al., 2012; Feng and Stockwell, 2018; Tao et al., 2020). The usual characteristics of ferroptotic cells include bulged mitochondria, diminished cristae, diminished mitochondrial membrane potential, and increased mitochondrial membrane permeability (Dixon et al., 2012), designating mitochondrial dysfunction.

Biochemically, iron is crucial for the normal cellular processes of organisms, such as DNA and energy production. Iron plays a part in the circulation of blood, where it forms a complex with the glycoprotein transferrin to form di-ferric transferrin, which eventually binds to its receptor (transferrin receptor 1, TFR1) on the surface of the cell (Abbaspour et al., 2014; Camaschella et al., 2020). This binding causes a conformational change in the receptor, bringing about endocytosis. The acidic environment within the vesicle causes an alteration in the structure of transferrin, leading to the release of one of its iron ions (Fe^2+^) (Chen et al., 2022). The released ion (Fe^2+^) is further transported across the vesicle membrane into the cell’s cytoplasm by a divalent metal transporter 1 (DMT1, also known as SLC11A2) (Chen et al., 2022). As a result, the transferrin receptor returns to the cell surface, where it can bind to more transferrin and repeat the process. Iron homeostasis is maintained during a cell’s normal physiological state, and an extra amount of Fe^2+^ is stored as ferritin, preventing over-accumulation (Bogdan et al., 2016). However, dysregulation of this process initiates the Fenton reaction, activating the generation of a huge quantity of free radicals, resulting in cell fatality and a general oxidative impairment of tissues (Chen et al., 2022). Ferritin can also be degraded via enzymatic reactions by a process known as ferritinophagy, which produces free Fe^2+^ that contributes to ferroptosis (Hasan et al., 2023). The deactivation of cystine-glutamate antiporter, SLC7A11, which leads to the build-up of ROS, facilitates ferroptosis. In addition, the induction of various white blood cells (neutrophils, macrophages, and eosinophils) involved during the body’s fight against infection can generate ROS (Latunde-Dada, 2017). These reactive species also act as intracellular messengers during signaling and cell death. Under a normal physiological state, these reactive species are balanced by antioxidants. However, an imbalance brings about an increased generation of ROS, resulting in oxidative stress, which poses a threat to cellular macromolecules (DNA, lipids, and proteins) (Afolabi, Osikoya & Okafor, 2016; Xie et al., 2022).

Basically, the biological membrane is highly susceptible to ROS due to its ability to solubilize molecular oxygen, thus subjecting the membrane phospholipids to high attack by these reactive species (Su et al., 2019). Lipid peroxidation takes place via two mechanisms: enzymatic and non-enzymatic.

• The non-enzymatic mechanism involves iron-dependent lipid peroxidation and entails three phases of reactions, namely, initiation, propagation, and termination (Feng and Stockwell, 2018). The initiation phase involves the oxidation of the acyl chain of polyunsaturated fatty acids (PUFA) to produce a lipid radical (R•, carbon-centered) as well as the abstraction of its hydrogen by hydroxyl (OH). In the propagation phase, the lipid radical forms a peroxyl radical (R-OO•) with oxygen; the peroxyl radical undergoes further propagation to form lipid hydroperoxide (R-OOH) by the abstraction of hydrogen from a phospholipid molecule. An additional phospholipid (bis-allylic position) can also form the R-OO-R • dimer with the peroxyl radical (R-OO•). During the Fenton reaction, alkoxyl radicals (RO•) can be produced by oxidatively cleaving lipid hydroperoxide (R-OOH). This mechanism of lipid peroxidation gives rise to various electrophiles (such as malondialdehyde). In the termination phase, the chain reaction is aborted by the reaction of the peroxyl radical with an antioxidant (such as α-tocopherol) or by the reaction of two lipid radicals to form a stable product (Feng and Stockwell, 2018).
• However, enzymatic lipid peroxidation is catalyzed by enzymes like lipooxygenase (LOX), cyclooxygenases, and cytochrome p450. LOX plays a part in the generation of R-OOH. An extremely ordered oxygenation center is involved in lipid peroxidation, and only one type of phospholipid is affected by oxidation (Feng and Stockwell, 2018). The most ubiquitous substrates for lipoxygenase are arachidonic and linoleic acids, which form hydroperoxyl groups with molecular oxygen at various carbon sites of acyl chains.

### 2.2 Signaling pathways in ferroptosis

#### 2.2.1 GPX4 and ferroptosis

**FIGURE 1:**
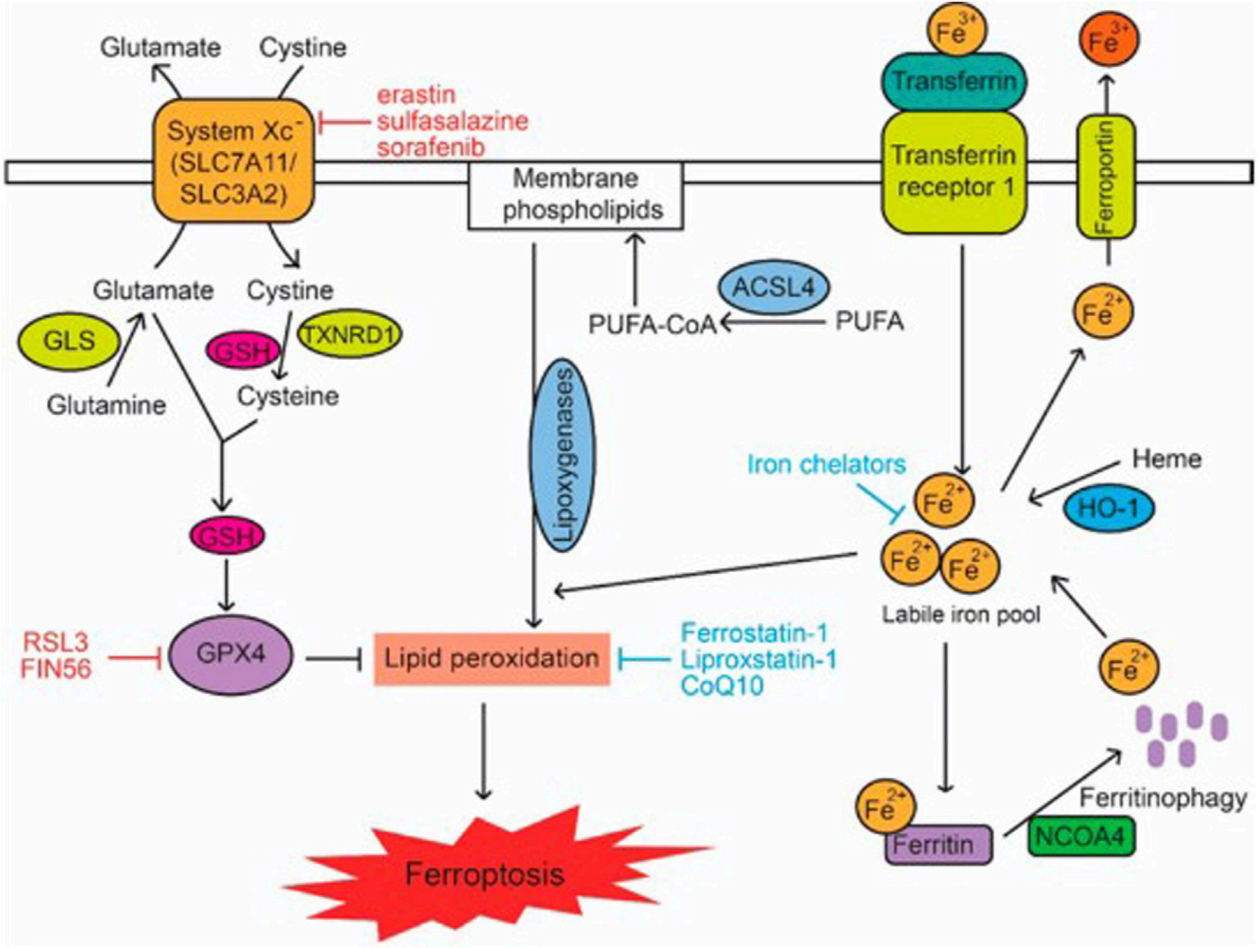
Signalling Pathways in Ferroptosis. Lipid peroxidation in cells is modulated by metabolic pathways including cystine and glutamine metabolism, GSH, PUFAs, and iron, which in turn regulate ferroptosis. The terms CoQ10, GLS, GSH, PUFA, and PUFA-CoA refer to glutaminase, polyunsaturated fatty acid, and PUFA-coenzyme A, respectively (Liu et al., 2020).

The antioxidant enzymes glutathione peroxidase 4, GPX4, and glutathione (as a cofactor) defend cells and membranes in opposition to peroxidation by preserving the fluidity of the membrane and offsetting lipid peroxides (Gan, 2021; Rotimi et al., 2019). Reduced glutathione (GSH) is re-cyclable by the successive reduction of oxidized glutathione disulfide (GSSG) by glutathione reductase and NADPH/H^+^. The inhibition and increased expression of GPX4 can lead to elevated and reduced ROS, respectively, wherein the latter averts ferroptosis (Jelinek et al., 2018; Kinowaki et al., 2018). Glutathione peroxidase 4, which can be deactivated either directly or indirectly by glutathione exhaustion, is a distinct and main regulator of ferroptosis (Conrad and Friedmann Angeli, 2015). An example of a specific GPX4 inhibitor is RAS-selective lethal 3 (RSL3). This inhibition process results in the production of ROS and thereby activates ferroptosis (Yang et al., 2014; Kim et al., 2023). A mevalonate pathway product, ubiquinone (CoQ10), acts as a free radical scavenger to inhibit ferroptosis in the membrane (Stockwell et al., 2017). FIN56, an activator of ferroptosis, exhausts CoQ10 by altering the activity of squalene synthase (SQS), which partially steers the build-up of lipid peroxidation. In addition, statin therapies and inhibitors of HMG-CoA reductase (a mevalonate pathway enzyme) stimulate ferroptosis, apparently by CoQ10 depletion and perhaps by the downstream inhibition of tRNA isopentenylation via tRNA isopentenyl transferase 1 (TRIT1), an essential for GPX4 synthesis (Stockwell et al., 2017) (Figure 1).

#### 2.2.2 System Xc and ferroptosis

The antioxidant ability of GPX4 is representative of its catalytic effect on glutathione (GSH) (Zhang et al., 2021). Hence, GSH serves as an indirect effector of the activity of GPX4. The amino acids cysteine, glycine, and glutamate are crucial for synthesizing GSH, a reaction catalyzed by glutamate cysteine ligase (GCL) and glutathione synthetase (Su et al., 2019). This antioxidant is imperative for the homeostatic regulation of redox reactions (Jia et al., 2020). Hence, GSH synthesis is influenced by the availability of these amino acids and enzymes. Cysteine-glutamate antiporter inhibition lowers the cysteine intracellular pool, impacting GSH levels (Kang et al., 2021). A viable constituent of the plasma membrane transporter, cysteine/glutamate antiporter solute carrier family seven-member 11 (SLC7A11), is charged with the exchange of internal and extracellular glutamate and cysteine, respectively (Koppula et al., 2018). Erastin, sorafenib, and sulfasalazine are all ferroptosis inducers (Wang et al., 2021). Erastin disrupts the transfer of cysteine, which is needed for glutathione synthesis, and also functions to inhibit system Xc selectively (Dixon et al., 2014; Sato et al., 2018). Similarly, sulfasalazine inhibits the cysteine/glutamate antiporter, which is crucial for regulating the activity of GPX4; hence resulting in the accumulation of lipid reactive oxygen species (Kim et al., 2018; Liu et al., 2022). In addition, sorafenib promotes ferroptosis by suppressing the activity of system Xc either by reducing SLC7A11’s transcription or decreasing the levels of glutamate in the cell (Li et al., 2023). Glutamate reduces the intracellular quantity of cysteine, interfering with ferroptosis. Glutamate silences the cysteine transporter, SLC7A11, via the activation of oxidative stress, resulting in the exhaustion of the glutathione concentration of the cell (Su et al., 2019).

#### 2.2.3 Nuclear factor erythroid-2-related factor 2 (NRF2)

NRF2 is a prominent transcription factor with an antioxidant role (Rojo de la Vega et al., 2018). Phase II detoxifying enzymes (such as glutathione S-transferase, UDP-glucuronosyltransferase, GPX4, glutathione reductase, and glutamate-cysteine ligase (GCLc and GCLm subunits), multidrug resistance-associated transporters (SLC7A11), thioredoxin 1, and NAD (P)H quinone oxidoreductase 1) are downstream genes of NRF2 (Song and Long, 2020). Hence, NRF2 has a key impact on ferroptosis regulation. Kelch-like ECH-associated protein 1 (Keap1) strictly controls the activation of NRF2. Keap1 actively targets NRF2 for ubiquitination and proteasomal destruction, in addition to passively isolating NRF2 from the cytoplasm. Under normal circumstances, NRF2 binds to Keap1 and is further deactivated by ubiquitination and proteasome destruction. However, when the cell is under the effects of oxidative stress or in the presence of numerous electrophiles or substances that are toxic to the cell, NRF2 is freed from the Keap1 binding site and quickly transported to the nucleus, where it interacts with the antioxidant response element (ARE) in the target gene’s promoter region and promotes transcription to control redox cellular homeostasis and oxidative stress balancing (Song and Long, 2020). In ferroptosis, NRF2 targets genes that code for the iron metabolism-related genes (TFR1, FPN, ferritin heavy chain 1 (FTH1), and ferritin light chain (FTL); heme metabolism-related genes [HO-1, ATP-binding cassette subfamily B member 6 (ABCB6)]; and solute carrier family member 48 member A1 (SLC48A1) (Tao et al., 2020). For instance, during malaria parasite’s lifecycle, they degrade the host red blood cells for their development and replication. This process results in the release of heme within the infected red blood cells, the accumulation of which facilitates oxidative stress and cellular damage. However, the host’s response to this infection involves the detoxification of heme by heme oxygenase-1 (HO-1) into ferrous iron and biliverdin following NRF2 activation, consequently mitigating the oxidative damage caused by malaria infection. ABCB6 plays a crucial role in heme’s synthesis, while SLC48A1 transports it. As a result, NRF2 promotion can repress iron intake, boost iron storage, reduce electrophiles, and guard against ROS and ferroptosis (Kerins and Ooi, 2018; Dodson et al., 2019). Interestingly, during NRF2’s activation, its target genes are either upregulated or downregulated, which depicts the concerted effort to tackle oxidative stress and maintain cellular balance. The upregulated genes include FPN, HO-1, and ABCB6, while FTH1 and FTL are downregulated (Yan et al., 2023). Another target of NRF2 is SLC7A11, a crucial part of system Xc, which is upregulated under NRF2 activation (Xu et al., 2019).

#### 2.2.4 Dual role of tumor suppressor protein 53 (TP53) in ferroptosis

In response to various stress signals, p53 is activated. This protein plays a role in various processes, such as cell cycle repression, senescence, and programmed cell death, as well as in ferroptosis (Tao et al., 2020). P53 genes directly stimulate the initiation of iron detectors (such as iron regulatory hormone) to control intracellular iron concentration. Of note, the promoter region of hepcidin antimicrobial peptide (HAMP), the gene that codes for hepcidin (a regulator of iron metabolism), is composed of a p53-responsive element and thus can be activated by p53, whereas its expression decreases when p53 is repressed (Weizer-Stern et al., 2007; Zhang and Chen, 2019). To control iron homeostasis in the mitochondria, a wild-type p53 reproduces the iron-binding protein XN (frataxin) present in the mitochondria (Shimizu et al., 2014). Moreover, FDXR/p53 loop formation allows p53 to promote the synthesis of iron oxidoreductase (FDXR), which avoids mitochondrial iron overaccumulation. Conversely, the FDXR deficit prevents the translation of p53 mRNA (Liu et al., 2020; Xu et al., 2023). In addition, the protein ferritin is made up of two subunits (light, FTL, and heavy chain, FTH1). p53 can enhance the translation of *fth1* mRNA as well as revoke the stability of transferrin-mRNA, thus resulting in an increase and decrease in cellular iron storage and import, respectively (Laubach et al., 2021; Xu et al., 2023). The upregulation of the heavy chain (FTH1) by p53 breaks the equilibrium between both subunits, hence ridding ferritin of its balance. Also, it was discovered that p53 cooperates with hypoxia-inducible factor 1α (HIF-1α) to boost p53 expression and stability in an iron-deficient state (Xu et al., 2023). Overaccumulation of iron can lower p53 protein levels and function. To prevent p53 from interacting with DNA, iron porphyrin heme binds to p53 directly, promoting p53’s nuclear export and destruction. Furthermore, via various processes, p53 also participates in the transcription of genes related to lipid metabolism. It can bind directly to the promoter area of sterol regulatory element-binding transcription factor 1 to suppress that gene’s expression, consequently controlling the expression of several genes that participate in lipid metabolism (Liu et al., 2019). Carnitine palmitoyl transferase 1C and phosphatidylate phosphatase are fatty acid oxidation-related enzymes whose transcription is controlled by p53. These enzymes control the movement of triggered fatty acids to the mitochondria, promoting the oxidation of fatty acids in cells (Liu et al., 2020; Xu et al., 2023). Malonyl CoA decarboxylase, which catalyses the oxygenation of intracellular fatty acids to avoid lipid build-up in cells, is another gene that p53 stimulates its transcription (Liu et al., 2019). In H1299 cells having their p53 gene repressed and subsequently exposed to ROS, it was discovered that the cellular activities remained unaltered. Contrariwise, 90% of fatality occurred in the cells when exposed to ROS following p53 activation (Kang et al., 2019). This fatality level reveals that these cells’ free radical scavenging ability was decreased by p53 activation. Moreover, the rate of cell death was lowered following treatment with the ferroptosis inhibitor fer-1. This further shows that p53 plays a significant role in ferroptosis. Also, p53 inhibits SLC7A11 to decrease cystine absorption and intracellular glutathione synthesis, which raises intracellular ROS (Liu et al., 2019; Li et al., 2020). In addition, the glutamine metabolic pathway is a potent regulator of ferroptosis. Glutaminases (GLS) 1 and 2 convert glutamine to glutamic acid during glutamine catabolism and are further converted into α-ketoglutarate, a crucial Kreb’s cycle substrate (Lieu et al., 2020). p53 activates GLS2 (present in the mitochondria) transcription and consequently controls how cells use oxygen and produce ATP (Xu et al., 2023). GSH and NADH synthesis in cells can be increased by GLS2, which can also activate antioxidant activity (Kang et al., 2019; Liu et al., 2020). Another crucial member of the lipoxygenase family that positively controls p53 and facilitates ferroptosis is ALOX12. Independent of GPX4 and ACSL4 initiation, ALOX12 repression can reduce p53-mediated ferroptosis caused by reactive species (Chu et al., 2019).

**FIGURE 2:**
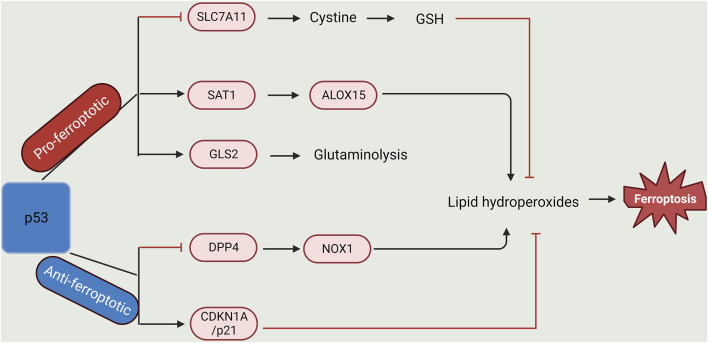
Dual-regulatory role of p53 in Ferroptosis. p53 plays a dual role in the regulation of lipid peroxidation in ferroptosis. On one hand, p53 can promote ferroptosis via the repression of SLC7A11 expression or activation of SAT1 and GLS2 expression. On the other hand, p53 could inhibit ferroptosis via the suppression of DPP4 activity or initiation of CDKN1A/p21 expression (Created with BioRender.com).

Apart from the promoting effect of p53, it can also repress ferroptosis. The p21 protein, a main p53 target that represses the destruction of glutathione, responds to stressors due to its ability to activate cell cycle interruption and aging (Kang et al., 2019). p53 transactivates p21 to prevent glutathione breakdown and to increase the activity of GPX4, which reduces ROS build-up from noxious lipids to prevent ferroptosis (Xu et al., 2023). Alternatively, p53 can repress ferroptosis by directly preventing DPP4 (dipeptidyl peptidase 4) activity or enhancing CDKN1A/p21 (cyclin-dependent kinase inhibitor 1A) expression. When p53 is deficient in the cell, DPP4 and NOX1 combine, forming a complex that promotes lipid peroxidation and ferroptosis (Tao et al., 2020) (Figure 2).

#### 2.2.5 Ferroptosis suppressor protein1 (FSP1)

This protein, previously referred to as flavoprotein apoptosis-inducing factor mitochondria-associated 2 (AIFM2), was retitled FSP1 following its anti-ferroptosis function (Bersuker et al., 2019; Doll et al., 2019). Ferroptosis suppressor protein 1 N-myristylation was reported to be crucial for its role, as mentioned earlier (Bersuker et al., 2019). In addition, ferroptosis resistance is enhanced by N-myristylation-dependent enrolment of FSP1 in the plasma membrane. Once enrolled, FSP1 inhibits lipid peroxides by degrading NAD(P)H and accelerating the conversion of ubiquinone (CoQ10) to its ubiquinol (CoQ10-H2). Furthermore, an acute decrease in cellular ubiquinone sensitises RSL3 to a lesser extent than the knockout of FSP1 when the ubiquinone synthesis enzyme CoQ2 is knocked out or inhibited with 4-chlorobenzoic acid (4-CBA) (Bersuker et al., 2019). This action pattern predicts that mechanisms involving FSP1 besides the FSP1-CoQ10-NAD(P)H pathway could also be responsible for ferroptosis inhibition (Zeng et al., 2022). Similarly, without the involvement of the ubiqunol pathway, FSP-1 hindered erastin-, sorafenib-, and RSL3-induced ferroptosis (Dai et al., 2020). Nevertheless, in FSP1-repressed cells, exogenously derived ubiquinone could not stop ferroptosis. Surprisingly, in both wild-type and FSP1-repressed cells, the expression of charged multivesicular body proteins 5 and 6 (CHMP5 and CHMP6) induced by RSL3 at the plasma membrane was inhibited by FSP1 knockdown, whereas CHMP5 overexpression prevented cell death caused by RSL3, erastin, and sorafenib (Zeng et al., 2022). In a recent screening of naturally occurring vitamin compounds, three classes of vitamin K (phylloquinone, menaquinone-4, and menadione) capable of shielding cells from GPX4-deletion-promoting ferroptosis were discovered (Mishima et al., 2022). Ferroptosis and neuronal ferroptosis are caused by ferroptosis inducers and glutamate, respectively, which are reversed by vitamin K classes. In contrast, other sets of regulated cell death, namely apoptosis, necroptosis, and pyroptosis, were not prevented (Zeng et al., 2022). Recent studies have shown that vitamin K effectively reduces the growth of lipid peroxides via a mechanism unrelated to its iron-chelating action (Mishima et al., 2022). These authors investigated whether FSP1 can mediate vitamin K reduction because vitamin K is a ubiquinone-like naphthoquinone with redox activity. *In vitro* incubations of recombinant human ferroptosis suppressor protein 1, NADH, and any of the three vitamin Ks resulted in the degradation of NADH and the synthesis of vitamin-hydroquinone. These show that FSP1 plays a vitamin K reductase role via the generation of its equivalent hydroquinone (VK-H2) to repress lipid peroxidation at the expense of NAD (P)H (Zeng et al., 2022). The anti-ferroptosis activity of vitamin K in FSP1 mutant cells could be restored by overexpressing wild-type FSP1 and not the myristylation-deficient form of the protein, suggesting that N-myristylation of FSP1 is crucial for this function.

## 3 Association between mitochondria and ferroptosis

The various organelles of the cell may sense and control stress signals (López-Otín and Kroemer, 2021) and thus be instrumental in the control or happening of various types of regulated cell death, including ferroptosis (Chen et al., 2021). However, the mitochondria, being the power house of the cell, regulate several activities in the cell, including energy metabolism, iron, and calcium metabolism, the conveying of endogenous and exogenous clues to other organelles of the cell, and several other functions (Wu et al., 2021). All these processes fine-tune the action of ferroptosis inducers. Hence, these and lots more are discussed in this section.

### 3.1 Mitochondrial membrane and ferroptosis

The functionality of the mitochondria depends on the integrity of their membrane. The induction of ferroptosis with RAS selective lethal 3 (RSL3) resulted in modifications in the morphology of the mitochondria as well as the clustering of fragmented mitochondria around the nucleus (Wang et al., 2020a). In addition, the oxidation of lipids takes place on the plasma membrane and the membranes of various organelles with the inclusion of mitochondria (Chen et al., 2021; Guo et al., 2022).

#### 3.1.1 Mitochondrial membrane lipids and ferroptosis

Phospholipids, sterols, and sphingolipids make up the major constituents of the membrane lipids (Klug and Daum, 2014). Phospholipids are naturally amphipathic, with a hydrophobic chain (constituted of a saturated or unsaturated fatty acyl group) at one end and a hydrophilic chain (containing phosphatidylethanolamine and phosphatidylcholine) at the other end, particularly the head (Guo et al., 2022). Polyunsaturated fatty acids (PUFAs) are the preferential candidates for oxidative degradation during ferroptosis (Rodencal and Dixon, 2023). This oxidative degradation of the phospholipids of PUFA leads to alterations in their membrane structure and fluidity and possibly forming pores (water-loving) that alter the plasma membrane barrier (Boonnoy et al., 2017). Mitochondria are composed of elevated amounts of phosphatidylethanolamine, and there is a possibility of the foremost occurrence of lipid peroxidation on the outer or inner mitochondrial membrane (Guo et al., 2022). Various mitochondrial enzyme systems are involved in lipid metabolism, including acyl-coA synthetase long-chain family member 4 (ACSL4) and lysophosphatidylcholine acyltransferase 3 (LPCAT3), which are leading factors in ferroptosis. ACSL4 ligates long-chain polyunsaturated fatty acids, including arachidonic acid and adrenic acid, with coenzyme A. After that, the products are re-esterified into phospholipids by LPCAT3 (Doll et al., 2017; Zou et al., 2019; Guo et al., 2022). PUFA-phospholipids could be enzymatically and non-enzymatically oxidized by lipooxygenases (LOXs), cyclooxygenases (COXs), and cytochrome P450, as well as Fe^2+^ via the Fenton reaction (Wu et al., 2021). The acyl-CoA synthetase long chain (ACSL) family of proteins is confined to the endoplasmic reticulum as well as the outer mitochondrial membrane to mediate β-oxidation via the conversion of fatty acids to acyl-CoA, with the latter being a lipid metabolism intermediate product that plays a part in the synthesis and degradation of lipid and fatty acids, respectively. ACSL4 has been implicated in ferroptosis, as proven in a breast cancer cell line study where cell lines showing ACSL4 are responsive to ferroptosis (Doll et al., 2017).

#### 3.1.2 Mitochondrial membrane proteins and ferroptosis

The outer mitochondrial membrane is made up of a protein (voltage-dependent anion channel, VDAC), which constitutes largely the covering of the denser parts of the membrane surface (Najbauer et al., 2021). This protein, categorized as a porin, allows the influx and efflux of ions and metabolites across the outer mitochondrial membrane, as well as being a major channel for the exchange of ATP and ADP in and out of the mitochondria and maintaining calcium levels in the mitochondria (Guo et al., 2022). However, VDAC participation in ferroptosis was enumerated based on its association with mitoNEET (Lipper et al., 2019). The confluence of cell survival and death indicators, mediated by diverse ligands or proteins, is thought to occur in VDACs (Guo et al., 2022). The opening of this channel (VDAC) results in elevated mitochondrial reactive oxygen species and impaired function of the mitochondria, consequently resulting in the cell’s death (DeHart et al., 2018). RAS oncogene-accommodated cells with an increased level of VDACs are highly sensitized to erastin-induced ferroptosis. In contrast, inhibiting two isoforms of VDACs by RNA interference brings about resistance (Guo et al., 2022). Another outer mitochondrial membrane protein, FUNDC2 (FUN14 domain-containing 2, also known as HCBP6) (Ta et al., 2022), plays a key role in the control of platelet activation via an AKT-GSK-3β-cGMP axis (Ma et al., 2019) and in the fragmentation of mitochondria by the suppression of mitofusin-1 (MFN1) (Li et al., 2022). Ta et al. (2022) reported that FUNDC2, in association with the mitochondrial amino acid transporter SLC25A11, negatively controls mitochondrial reduced glutathione levels and controls ferroptosis. The association between FUNDC2 and SLC25A11 is improved under the activation of ferroptosis, which subsequently lessens the dimerization of SLC25A11, hence leading to a reduction in the level of reduced glutathione (GSH) in the mitochondria and consequently lipid peroxidation and ferroptosis. The FUNDC2-SLC25A11 axis is a recently discovered mechanism in mitochondria that controls ferroptosis (Guo et al., 2022).

### 3.2 Energy metabolism of mitochondria and ferroptosis

#### 3.2.1 Oxidative phosphorylation and ferroptosis

The mitochondria’s major role entails generating energy for cellular processes. The cells are the major energy source for various processes of oxidative phosphorylation, with the exclusion of cancer cells, which solely rely on glycolysis (Guo et al., 2022). The major rate-limiting enzymes of the glycolytic process in cancer cells, namely, hexokinase II, phosphofructokinase, and pyruvate kinase M2, have been reported to be suppressed following treatment with a ferroptosis inducer (RSL3) in glioma cells (Wang et al., 2020a). Diverse enzymes that participate in mitochondrial respiration are also involved in controlling ferroptosis, including fumarate hydratase, aconitase, and cytochrome c oxidase II (Gao et al., 2019). Cells void of mitochondria are sensitive to substances that induce ferroptosis and receptive to ferroptosis induced by the inhibition of cysteine (Wang et al., 2020a). Most metabolites enter the mitochondria via the opening of VDACs, elevating the metabolism of the mitochondria, resulting in the production of reactive oxygen species and, after that, impaired function of the mitochondria (Wang et al., 2020a). The main location of reactive oxygen species (ROS) generation is the mitochondria. ROS are generated as superoxide in the electron transport chain, which is strictly controlled (Guo et al., 2022). Cells are rescued from lipid peroxidation-induced impairment following treatment with free radicals’ scavengers targeted at the mitochondria. For instance, Fang et al. reported that mitoTEMPO, a mitochondria-targeted antioxidant, averted ferroptosis stimulated by doxorubicin in cardiomyopathy, suggesting mitochondria as a key player in ferroptosis induced by doxorubicin in heart disease (Fang et al., 2019). Pyrimidine bases are essential for cell multiplication and are produced by uridine monophosphate generated from the *de novo* biosynthesis of pyrimidine in the mitochondria, where dihydroorotate dehydrogenase (DHODH) catalyzes the conversion of dihydroorotate to orotate. This enzyme connects the electron transport chain through the Coenzyme Q pool and requires Coenzyme Q as an electron acceptor (Boukalova et al., 2020). Dihydroorotate dehydrogenase can mediate the reduction of ubiquinone to ubiquinol in the mitochondria, which preserves the cells from ferroptosis by the detoxification of lipid peroxides (Tadokoro et al., 2020), similar to suppression of ferroptosis by the action of mitochondrial glutathione peroxidase, and occurs irrespective of the cytosolic presence of glutathione peroxidase or ferroptosis suppressor protein 1 (Mao et al., 2021).

### 3.3 Mitochondrial amino acid metabolism, calcium, and ferroptosis

In a normal physiological state, glutamine and transferrin are essential for the growth and viability of the cell, whereas their deficiency induces ferroptosis (Guo et al., 2022). Similarly, cysteine is necessary for synthesising reduced glutathione to maintain the redox state and is converted to some biologically active molecules, including iron-sulfur clusters (Guo et al., 2022). Due to cysteine deficiency, Mitochondria play a key role in ferroptosis induction (Gao et al., 2019). However, the suppression of cysteine that induced lipid peroxidation and ferroptosis was attenuated via the suppression of the mitochondrial TCA cycle or electron transport chain.

Accumulation of reactive oxygen species and an elevated calcium level in the cell will result in possible membrane collapse, increased calcium, and fracture of the mitochondria (De Nicolo et al., 2023; Iheagwam et al., 2021). As reported by Maher et al. (2018), suppression of the toxic effect of glutamate by the blockage of mitochondrial reactive oxygen species generation or decrease of calcium inflow can preserve cells from ferroptosis-inducers such as erastin, sulfasalazine, or any cysteine/glutamate antiporter inhibitor.

### 3.4 Mitochondrial iron metabolism and ferroptosis

#### 3.4.1 Mitochondrial iron and ferroptosis

The cells uptake extracellular iron and transport it into mitochondria through the mitochondrial iron importer solute carrier family 25 member 37 (SLC25A37) and solute carrier family 25 member 28 (SLC25A28), also known as mitoferrin-1 and mitoferrin-2, respectively (Chen et al., 2021). Mitochondrial iron (II) synthesises heme and iron-sulfur clusters or preserves them in mitochondrial ferritin. Contrariwise, accumulated mitochondrial iron promotes ROS generation, gives rise to anomalous enzymatic activity, and further promotes ferroptosis (Guo et al., 2022). Primary neurons or human monocytic cells can directly undergo ferroptosis in the presence of heme. This process can then be dual-controlled by cytosolic or mitochondrial heme oxygenase 1 (HMOX1), depending on the kind of cell (Adedoyin et al., 2018; Chen et al., 2021). The NEET proteins are encoded by three genes in humans. The outer mitochondrial membrane-confined protein mitoNEET, also known as CDGSH iron sulphur domain 1 (CISD1), is necessary for the control of iron metabolism, the functionality of the membrane, and reactive oxygen species maintenance (Guo et al., 2022). The misplacement of CISD1 brings about the build-up of iron in the mitochondria and oxidative damage, eventually leading to the induction of ferroptosis by erastin in neoplastic cells (Guo et al., 2022). Also, repression of the CISD2 gene can evade head and neck cancer resistance to ferroptosis by sulfasalazine via the elevation of lipid peroxides and iron in the mitochondria (Kim et al., 2018). However, CISD3 played a key role in ferroptosis induced by the inhibition of cystine. CISD3 exhaustion brings metabolic diversion to glutaminolysis, activating ferroptosis (Li et al., 2021b).

#### 3.4.2 Iron-sulfur (Fe-S) clusters and ferroptosis

Iron-sulfur clusters are imperative cofactors for numerous proteins that play a key role in the production of lipids and energy, as well as iron metabolism and DNA preservation, and they are primarily generated in the mitochondria (Ward and Cloonan, 2019). During the synthesis of Fe-S clusters, they are initially generated on a complex made up of two proteins, iron-sulfur cluster assembly enzyme (ISCU) and ISD11, as well as an NFS1 cysteine desulfurase enzyme in the mitochondria, necessitating electrons, cysteine, and iron (Guo et al., 2022). Immediately after their synthesis, [2Fe-2S] clusters are liberated from the iron-sulfur cluster assembly enzyme and relayed to glutaredoxin-related protein 5. After that, [2Fe-2S] clusters are intercalated into [2Fe-2S] proteins directly or [4Fe-4S] clusters tardily (Guo et al., 2022). Furthermore, these clusters are transported out of the mitochondria to the cytosolic space by the iron-sulfur cluster export machinery ABCB7 (Wachnowsky et al., 2018). During the initial phase of iron-sulfur cluster production, iron and sulfur are brought together into the Fe-S clusters, which are liberated by NFS1 cysteine desulfurase from cysteine. A limited amount of sulfur incapable of mediating the NFS1 reaction will lead to the accumulation of free labile iron in the mitochondria (Guo et al., 2022). NFS1 is essential to the homeostasis of Fe-S clusters. When the iron-sulfur clusters are limited, it activates the iron-deficient response, which triggers ferroptosis along with glutathione expenditure (Alvarez et al., 2017). In addition, when NFS1 is repressed, it consequently initiates the iron-responsive element binding protein 2 (IREB2/IRP2)-mediated iron-deficient response and makes lung cancer cells responsive to ferroptosis (Chen et al., 2021; Terzi et al., 2021). Furthermore, iron-sulfur cluster assembly enzymes (ISCU) participate maximally in the synthesis of iron-sulfur clusters. The excessive expression of ISCU suppresses dihydroartemisinin-induced ferroptosis via the preservation of the functionality of the mitochondria, elevation of GSH levels in the cell, and iron metabolism control (Du et al., 2019). Frataxin (FXN) is confined to the matrix of the mitochondria and functions by donating iron to ISCU for [2Fe-S] clusters synod, as well as controlling the synthesis of sulfur (Guo et al., 2022). Alteration in mitochondrial function, elevated oxidative stress, and iron accumulation are associated with FXN reduction, and all these make cells responsive to the ferroptosis-inducer erastin (Cotticelli et al., 2019). Hence, proteins that play a role in Fe-S cluster synthesis seem to function as protectants against ferroptosis.

## 4 Mitochondria-mediated ferroptosis in malaria

### 4.1 Evidence of a relationship between ferroptosis and malaria

#### 4.1.1 Host innate immune responses

The inherent antigen-presenting cells (APCs) of the immune system initiate the first guard against malaria parasites through the promotion of pattern recognition receptors (PRRs) through the identification of the molecular patterns in pathogen-related *Plasmodium* (such as the nucleic acids as well as glycosylphosphatidylinositol anchors) and molecular patterns that can be impaired (such as uric acid, hemozoin, and heme) (Gowda and Wu, 2018; Sena-dos-Santos et al., 2021). In the hepatocyte, the recognition of the parasite’s ribonucleic acid (RNA) through the mitochondrial antiviral signaling protein (MAVs) initiates a strain I interferon (IFN-I) response, which activates the action of cells that secrete cytokines as well as induce oxidative stress (Gowda and Wu, 2018). However, during the erythrocytic phase, recognition through the Toll-like receptors of glycosylphosphatidylinositol anchors, the parasite’s hereditary material, and molecular patterns that can be impaired in the infected erythrocytes will determine the engagement (Sena-dos-Santos et al., 2021). When the infection-fighting white blood cells, natural and T killer cells, dendrites, and inflammasomes are activated, as well as the production of inflammation-promoting cytokines and oxidative stress inducers, these will cause the infected and immune cells to go through cell death (Sena-dos-Santos et al., 2021).

#### 4.1.2 Artemisinin mechanism of action

All drugs that have artemisinin (ART) as one of their constituents undergo metabolism to produce dihydroartemisinin (DHA; an endoperoxide) (Lu et al., 2019). This derivative functions in parasite destruction by generating reactive species after degradation (Siddiqui et al., 2022). For ART activation to occur, the derivative (endoperoxide) bond goes through reductive splitting mediated by Fe^2+^ (Lu et al., 2019). The main source of the ferrous ion is the host’s hemoglobin, whose proteolytic process releases active heme products. Most heme is concealed as hemozoin clusters, leaving a minute quantity for artemisinin activation (Ma et al., 2021). Other sources of iron, such as the labile iron pool (low level) regulated in the parasites, may also activate ART. ART’s dependence on iron for its activation is the underlying mechanism of its action against erythrocytic-stage malaria parasites (Lu et al., 2019). Additionally, artemisinin’s mechanism of action against malaria parasites entails protein degradation by radicals during ART activation. These radicals induce vast damage to the cell by targeting a broad span of proteins, lipids, and membrane constituents. Bridgford et al. (2018) revealed that dihydroartemisinin, via the repression of proteasomes, induces stress, which synergistically damages proteins that are already in existence and alters the right folding of freshly produced ones. This multi-faceted action of ART on diverse proteins would primarily result in endoplasmic reticulum stress and translation attenuation, which results in the fatal accumulation of polyubiquitinated marred proteins. Another mechanism of dihydroartemisinin (DHA) against malaria parasites is glutathione metabolism-ferroptosis via depletion of glutamate (Li et al., 2022). In the same study, ferroptosis inducers in synergy with DHA efficaciously inhibited malaria parasites at the erythrocytic stage. However, deferoxamine (an iron chelator) and liproxstatin-1 had an opposing parasite-eliminating effect but were able to lessen the build-up of a labile iron pool in the cell and lipid ROS induced by dihydroartemisinin (Li et al., 2022). Hence, this confirms the ferroptosis-induced mechanism of action of artemisinin.

#### 4.1.3 Ferroptosis-mediated destruction of liver stage plasmodium

Kain et al. (2020) reported that the genetic or therapeutic alteration of the SLC7A11-GPX4 signaling pathway led to a notable decrease in parasite count in the hepatocytes of infected mice. They further discovered increased lipid peroxidation on the membrane of the parasite after erastin-mediated treatment, as well as a reduction in the NADPH oxidase 1/TFR1 regulatory mechanism. This study also affirmed that the non-apoptotic cell death involved in malaria parasite destruction at the hepatocyte by the action of p53 is ferroptosis. However, p53’s ferroptosis-induction ability has been discredited in African descent due to the presence of a SNP noticed at codon 47 of the TP53 gene (Leu et al., 2019). For instance, detecting a p53 Ser47 SNP in a population of African descent made the cancer cells more resistant to RSL3 treatment (Jennis et al., 2016). This polymorphism could be an additive factor in elevating the malaria menace in Africa. Singh et al. (2020) showed that polymorphism could be advantageous against parasites by producing a disrupted macrophage with reduced sensitivity to hemozoin. This peculiar characteristic of polymorphism could only lessen the symptoms’ acuteness and not the parasite’s viability (Amos Alvan et al., 2022). An investigation into how diet affects the host’s vulnerability to Plasmodium infection revealed that feeding mice with an elevated-fat meal for 4 days reduces *Plasmodium* liver-stage infection by more than 90% (Zuzarte-Luís et al., 2017). This research showed that, following an effective invasion, the sporozoites of the parasite were destroyed inside the liver cells. An investigation into the underlying mechanism revealed that oxidative stress was responsible because the antioxidant N-acetylcysteine eliminated the effect. However, their study did not evaluate the role of an elevated-fat diet in generating ferroptosis. Nevertheless, the involvement of ferroptosis is still considered a possibility.

### 4.2 Ferroptosis in malaria therapy via mitochondrial targeting

#### 4.2.1 Targeting ROS generation

The accumulation of iron in the mitochondria generates reactive species via the Fenton reaction, which in turn facilitates ferroptosis induction. The iridium (III) complex promotes ROS accumulation in cancer cells to induce ferroptosis by regulating the expression of heme oxygenase 1 (HMOX1) (Wang et al., 2020b). *In vitro* and *in vivo* administration of cyclometalated iridium (III) polypyridyl complexes against multidrug-resistant malaria parasites showed potent antimalarial activity via the accumulation of ROS in the parasite’s mitochondria and alteration of the mitochondria membrane potential (Kumari et al., 2023). Additionally, artemisinin and its derivatives, such as artesunate, induce the parasite’s quick membrane potential depolarisation (Gomes et al., 2022). This process suggests the potential of targeting malaria parasite mitochondria with ferroptosis inducers to develop novel and effective antimalarial therapies.

#### 4.2.2 Targeting dihydroorotate dehydrogenase (DHODH)

A mitochondrial enzyme, DHODH, takes part in the crucial pyrimidine biosynthesis pathway. Additionally, DHODH, in synergy with mitochondrial GPX4 inhibits ferroptosis in the inner mitochondria membrane via the reduction of ubiquinone to ubiquinol (Cheng et al., 2023). Recent antimalarial development efforts have advanced a DHODH-targeting DSM265 into clinical evaluation (Ke and Mather, 2017). This drug candidate demonstrated selective inhibitory activity against DHODH and impacted parasite growth at both the liver and blood stages of *P. falciparum* (Sulyok et al., 2017) Table 1.

**TABLE 1:** Mitochondrial-ferroptosis as antimalarial target.

S/N	Drug/Therapy	Target	Effect	References
1	Combined therapy (dihydroartemisinin and erastin/sorafenib)	Glutamate depletion	Parasitaemia control at the erythrocytic stage	Li et al. (2022)
2	Atovaquone	Cytochrome b complex	Disrupt the mitochondrial electron transport chain at the asexual stage	Fry and Pudney, 1992; Chen et al., 2024
3	AQ-13 (Phase II Clinical Trial)	Heme	Inhibition of heme detoxification; disrupt the membrane function; at the asexual stage	Koita et al., 2017; Mengue et al., 2019; Umumararungu et al., 2023
4	DSM256 (Phase II; NCT02123290)	Dihydroorotate Dehydrogenase (DHODH)	Disrupts the mitochondrial electron transport chain	Phillips and Rathod, 2010; Coteron et al., 2011
5	Cyclometalated iridium (III) polypyridyl complexes (Ir1-Ir12)	Increased ROS and mitochondrial membrane potential disruption	Antimalarial activity against *P. berghei* (asexual gametocyte stages) and *P. falciparum*	Kumari et al. (2023)
6	MMV693183 (Preclinical)	Acetyl-CoA synthetase	Potency against asexual blood stages of both *P. falciparum* and *P. vivax,* and against *P. falciparum* gametocytes	Forte et al., 2021; De Vries et al., 2022
7	MMV688533 (acylguanidines; Preclinical)		*P. falciparum* asexual blood stage	Murithi et al. (2021)

#### 4.2.3 Targeting glutathione (GSH)

Glutathione is an antioxidant that is one of the defense systems against ROS accumulation, whose loss triggers ferroptosis. Li et al. (2022) reported in their study that the mechanism of dihydroartemisinin (DHA) against the malaria parasite is related to glutathione metabolism-ferroptosis via depletion of glutamate. Summarily, targeting GSH reduction may be viable for ferroptosis induction in malaria parasites.

## 5 Conclusion and future perspectives

Ferroptosis is a special type of regulated cell death utilizing the “iron weapon” to destroy the cell, which entails diverse types of cellular metabolism, including iron, lipids, ROS, and amino acids. Mitochondria do not only function as “energy factories” that supply ATP for cellular processes but also as “cellular suicidal weapon stores” that control cell death. Mitochondria are the main source of reactive oxygen species, which may make cells susceptible to ferroptosis. In addition, they play a key role in iron, lipid, and end-energy metabolism, making them an ideal target for ferroptosis execution. Through the destruction of infected erythrocytes or hepatocytes or the eradication of parasites, ferroptosis may impact malaria pathogenesis. Elevation in the haem concentration by the action of the malaria parasite during the breakdown of hemoglobin may trigger the Fenton reaction, producing surplus lipid hydroperoxides. These hydroperoxides could saturate the antioxidant defense of the parasite, causing iron-dependent cell death. Mitochondrial-ferroptosis has recently been identified as a target for some of the existing (artemisinins, artesunate, etc.) and budding therapies with antimalarial activity Table 1.

Cell death pathways, including ferroptosis, are currently known to play a part in response to infections, following the direct linkage of the death of an infected cell to malaria immunopathology and parasite mortality. Hence, these pathways are implicated as inherent mechanisms of the immune system and a key process for comprehending host-parasite interaction. Ferroptosis was characterised not too long ago, and little is known about its impact on the biology and pathophysiology of malaria parasites. A lot is yet to be unraveled in the genetic, molecular, and biochemical mechanisms triggered in its pathway during *Plasmodium* infection. Although it has been shown that ferroptosis can limit parasitemia in the liver, little is known about its immune potential. It would be interesting for future investigations to uncover these to facilitate its role in the prevention or treatment of malaria.

While targeting mitochondria-mediated ferroptosis holds potential for antimalarial interventions, diverse limitations, and challenges need to be addressed. A vital limitation is specificity, owing to the ability of several ferroptosis-targeting drugs to trigger other types of cell death, resulting in side effects on normal cells. However, the malaria parasite mitochondrion is an essential organelle in the various lifecycle stages. Hence, finding the parasite analog of the molecular pathways linked to mitochondrial ferroptosis in humans may dispense new opportunities for the selective activation of iron-dependent cell death in the parasite while the host is spared. Furthermore, the cell membrane barrier limits the effectiveness of drug delivery to the mitochondria. Therefore, innovative drug delivery systems, such as nanomaterials, could be utilized to increase targeted drug delivery. If ferroptosis is extensively explored, the *Plasmodium* life cycle could be effectively apprehended at both the liver and erythrocytic phases. Following the evolutionary association of *Plasmodium* species to other apicomplexan parasites such as *T. gondii* and *Babesia*, ferroptosis holds a potential for their control.
